# Interference Assembly and Fretting Wear Analysis of Hollow Shaft

**DOI:** 10.1155/2014/919518

**Published:** 2014-05-12

**Authors:** Chuanjun Han, Jie Zhang

**Affiliations:** School of Mechatronic Engineering, Southwest Petroleum University, Chengdu 610500, China

## Abstract

Fretting damage phenomenon often appears in the interference fit assembly. The finite element model of hollow shaft and shaft sleeve was established, and the equivalent stress and contact stress were computed after interference assembly. The assembly body of hollow shaft and shaft sleeve was in whirling bending load, and the contact status (sticking, sliding, and opening) and the distribution of stress along one typical contact line were computed under different loads, interferences, hollow degrees, friction coefficient, and wear quantity. Judgment formula of contact state was fixed by introducing the corrected coefficient *k*. The computation results showed that the “edge effect” appears in the contact surface after interference fit. The size of slip zone is unchanged along with the increase of bending load. The greater the interference value, the bigger the wear range. The hollow degree does not influence the size of stick zone but controls the position of the junction point of slip-open. Tangential contact stress increases with the friction coefficient, which has a little effect on normal contact stress. The relationship between open size and wear capacity is approximately linear.

## 1. Introduction

In many conditions, hollow shaft needs interference fit with other parts in normal work. Interference fit is a common mechanical component, semipermanent assembly, often because of the elastic deformation amount of the difference between the assembly parts, resulting in the local area of the contact surface relative reciprocating motion of small amplitude, which causes fretting damage [1]. Fretting of the two contact surfaces is of very small amplitude, which usually occurs in approximation to the fastening contact surfaces in a vibrating environment. Fretting can cause friction and wear, component bite, loss, acceleration of the crack initiation, propagation, and reduction of the fatigue life of components. Therefore, fretting damage is known as the cancer in the industry [2]. Fretting wear is one of the main reasons for key components' failure and often reduces 20%∼50% of fatigue life for material [3].

The fretting phenomenon of interference is very complex. It is subject to the relative slip amplitude, contact stress, friction coefficient, tangential force, thrust force, and other factors of the mating surfaces. And it is difficult to measure these parameters in the test. With the development of computer technology, finite element analysis methods have been increasingly used for fretting study. Yang et al. studied the effects of casing length, thickness, friction coefficients and interference on the contact pressure, and friction shear force of the sleeve specimens by using finite element software ABAQUS [4]. Zeng et al. finished the numerical simulation analysis of the wheels' interference surfaces fretting in ANSYS and got the law of the relative slip of contact node pairs of the hub and seat [5]. Fadag et al. analyzed the effects of contact load, friction coefficient, and shear force on the crack propagation of aluminum alloy fretting model [6]. However, few scholars focus on the fretting damage analysis of the hollow shaft of interference or only analyses contact stress after interference fit without considering the actual working condition load, such as Liu just studied the interference fit elastic-plastic stress of the hollow roll, but not the fretting damage [7]. In finite element software ADINA, the interference fit fretting model of the hollow shaft was established; the fretting damage law affected by the rotating bending load, interference, hollow degrees, friction coefficient, and wear were studied.

## 2. Interference Fit Contact Model

Inner radius of cylinder is *r*_1_; outer radius is *r*_2_. When the cylinder only bears internal pressure *p*_1_, the displacement of radius *r* is

(1) $$\begin{array}{c} u_{1}=\frac{r_{1}^{2}p_{1}}{E\left(r_{2}^{2}-r_{1}^{2}\right)}\left[\frac{\left(1+\nu \right)r_{2}^{2}}{r}+\left(1-\nu \right)r\right], \end{array}$$

where *E* is elasticity modulus of the cylinder material and *ν* is Poisson's ratio.

When the cylinder only bears outside pressure *p*_2_, the displacement of radius *r* is

(2) $$\begin{array}{c} u_{2}=-\frac{r_{2}^{2}p_{1}}{E\left(r_{2}^{2}-r_{1}^{2}\right)}\left[\frac{\left(1+\nu \right)r_{1}^{2}}{r}+\left(1-\nu \right)r\right]. \end{array}$$

**Figure 1:**
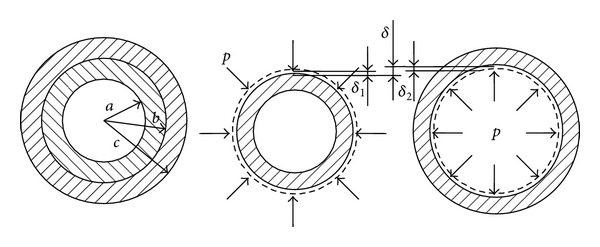
Assembly diagram of shaft and sleeve.

As shown in Figure 1, supposing inner radius of the hollow shaft is *a*, outer radius is *b* + *δ*_1_. Inner radius of the shaft sleeve is *b* − *δ*_2_, and outer radius is *c*. The interference is

(3) $$\begin{array}{c} \delta =\delta_{1}+\delta_{2}, \end{array}$$

where *δ*_1_ is the radial displacement of the hollow shaft outer surface and *δ*_2_ is the radial displacement of the shaft sleeve inner surface.

The contact pressure *p* will be produced on the contact surface by making use of temperature difference method to finish the assembly of hollow draft and the shaft sleeve. Then, according to (1) and (2), *δ*_1_ and *δ*_2_ are [8]

(4) $$\begin{array}{c} \delta_{1}=\frac{pb}{E_{1}}\left(\frac{b^{2}+a^{2}}{b^{2}-a^{2}}-\nu_{1}\right),\\ \delta_{2}=\frac{pb}{E_{2}}\left(\frac{c^{2}+b^{2}}{c^{2}-b^{2}}+\nu_{2}\right), \end{array}$$

where *E*_1_, *E*_2_ are the hollow shaft's and shaft sleeve's elastic modulus and *υ*_1_, *υ*_2_ are the hollow shaft's and shaft sleeve's Poisson ratio. Equations (4) should be substituted into (3) to yield

(5) $$\begin{array}{c} p=\left(\delta \right)\times {\left(\frac{b}{E_{1}}\left(\frac{b^{2}+a^{2}}{b^{2}-a^{2}}-\nu_{1}\right)+\frac{b}{E_{2}}\left(\frac{c^{2}+b^{2}}{c^{2}-b^{2}}+\nu_{2}\right)\right)}^{-1}. \end{array}$$

If *E*_1_ = *E*_2_ = *E*, *υ*_1_ = *υ*_2_ = *υ*, then

(6) $$\begin{array}{c} p=\frac{E\delta \left(b^{2}-a^{2}\right)\left(c^{2}-b^{2}\right)}{2b^{3}\left(c^{2}-a^{2}\right)}. \end{array}$$

## 3. Computational Model

**Figure 2:**
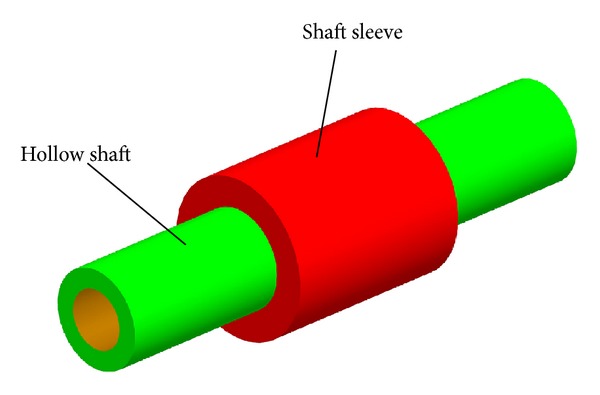
Assembly model of hollow shaft and bush.

Figure 2 shows the interference fit model of hollow shaft and shaft sleeve. The hollow shaft length is 50 mm, outer diameter is 10 mm, and inner diameter is 6 mm. Shaft sleeve's total length is 20 mm, outer diameter is 16 mm, and inner diameter is 9.988 mm. The materials of shaft and shaft sleeve are both alloy steel, the material's elastic modulus *E* = 201 GPa, Poisson's ratio *υ* = 0.3, and density *ρ* = 7800 kg/m^3^.

**Figure 3:**
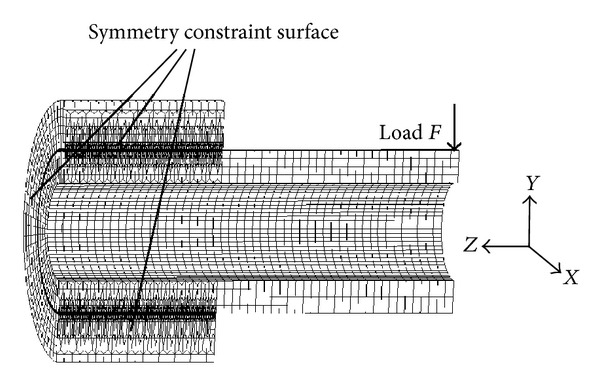
Finite element model of hollow shaft and bush.

Because of the symmetry model of the geometry and load, we take 1/4 of the original structure to build the finite element model with 8-node hexahedral meshing element, which is shown in Figure 3. Symmetry constraints are applied on the *XY* and *YZ* cross-section of the finite element model, respectively. The entire calculation process is divided into two load steps, the first step is to assemble the model and achieve true interference contact between the two parts, and the interference is 0.006 mm. The second step is to impose fixed constraints on the outer surface of the shaft sleeve and apply the bending load *F*  (1500 N) at the rightmost node of the shaft.

## 4. Interference Fit Analysis

**Figure 4:**
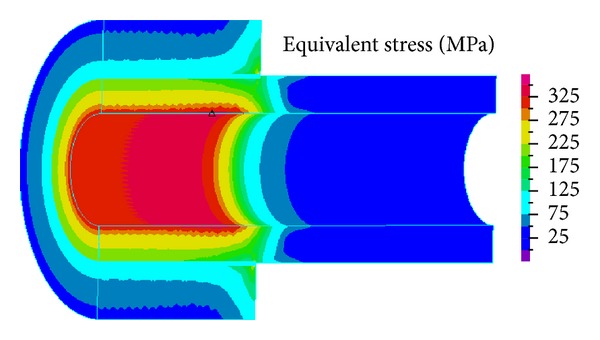
Equivalent stress of the assembly body.

**Figure 5:**
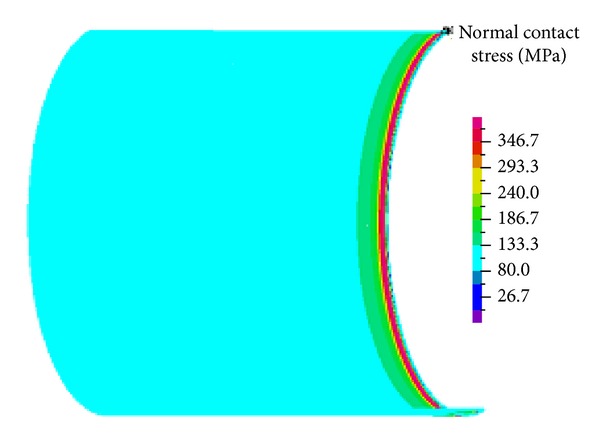
Normal contact stress of the contact surface.

Figure 4 shows the von Mises stress of the hollow shaft and shaft sleeve after interference assembly. The maximum stress of the hollow shaft is in the inner surface of interference fit period. Along the radial direction from inside to outside, stress decreases gradually, and the maximum stress is 339 MPa. There is a stress concentration phenomenon in the inner surface collar end of shaft sleeve with the maximum stress value 327.4 MPa, and the stress of shaft sleeve is also gradually reduced along the radial direction.  Figure 5 shows the normal contact stress distribution on the contact surface. The contact stress in the contact section end appeared “edge effect,” but the distribution of the contact stress in the contact section intermediate parts is more uniform.

**Figure 6:**
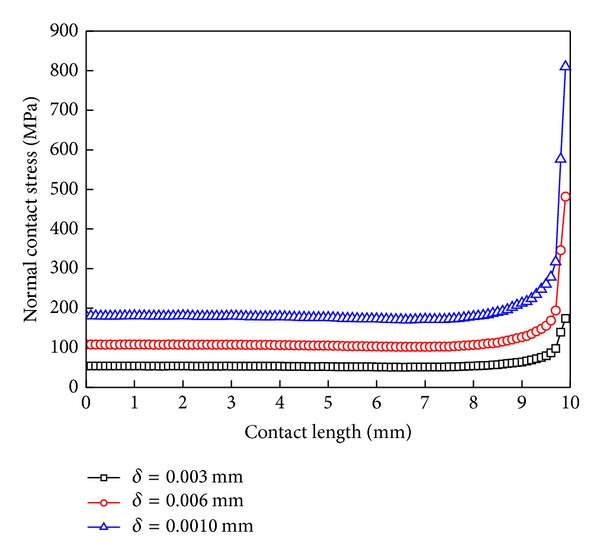
The normal contact stress distribution under different interference values.

Figure 6 shows the normal contact stress distribution on the contact surface, when the interferences are, respectively, 0.003 mm, 0.006 mm, and 0.01 mm. The greater the interference is, the greater the normal contact stress is, but the change and interference have no linear relationship. Interference increase also makes contact stress's “edge effect” more apparent. The maximum contact stress value is 809.9 MPa when the interference is 0.01 mm.

**Figure 7:**
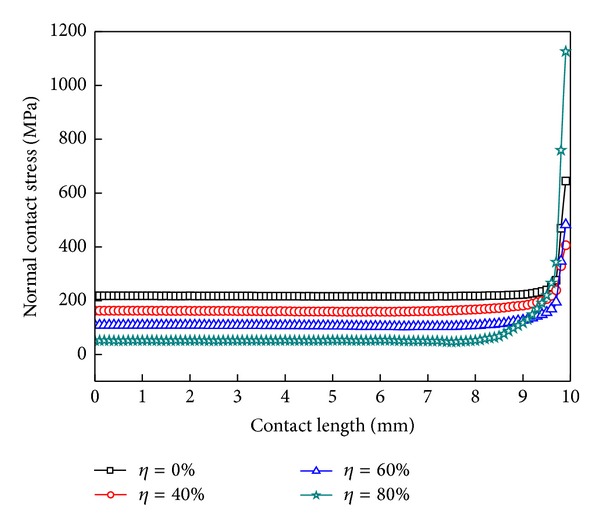
The normal contact stress distribution under different hollow degrees.

By defining the hollow degree of the shaft as the ratio of the inner radius and outer radius, the hollow degree influences the shaft's stiffness and deformation directly.  Figure 7 shows the normal contact stress distribution with the same interference, when the hollow degree is, respectively, 40%, 60%, and 80%. The greater the hollow degree of the shaft is, the smaller the contact stress is from the whole, and the solid shaft has the maximum contact stress. The greater the degree of hollow shaft is, the more obvious the “edge effect” is. The axial end maximum contact stress value of the solid shaft is greater than the shaft of hollow degree 40% or 60%, but less than the 80%.

## 5. Fretting Wear Analysis

The stress state of the contact zone interference is the main controlling factor of fatigue crack initiation and propagation. Actually fretting cracks firstly appear in stick-slip zone at first, and the local shear stress and the alternating macroscopic axial stress lead to the initiation of inclined cracks. Under the alternating external loads, the junction point of the stick-slip on the contact surface moved, and the inner alternating tensile stress distribution controls early expansion of microcracks. So, fretting contact stress distribution analysis is the key point of fretting fatigue [3].

**Figure 8:**
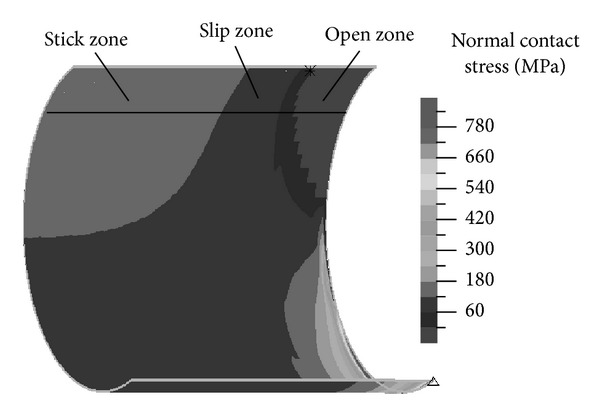
Normal contact stress distribution.

Figure 8 shows the normal contact stress on the contact surface when the friction coefficient is 0.2. It can be seen that the upper contact surface generates three regions, namely, stick zone, sliding zone, and open zone under the bending load. In the stick zone, wear will not occur between the contact surfaces. Stick wear, fatigue wear, and abrasive wear are easy to occur in slip zone and open zone mainly generates corrosive wear [9].

Because the hollow shaft does not carry the axial external loads, the shaft sleeve fretting amplitude is mainly caused by the deformation from the bending load, and, thus, the contact surfaces' contact state criterion needs to be revised. According to the simulation results, we have introduced a correction factor *k* related with the magnitude of interference, the hollow degrees of the shaft, and the moment load parameters. When |*σ*_*τ*_| < *kμ*|*σ*_*n*_|, the shaft sleeve inner surface and the shaft surface have no relative slip, which is corresponding to the slip zone in Figure 8; when |*σ*_*τ*_| ≥ *kμ*|*σ*_*n*_|, relative slip appears on the contact surface, which is corresponding to the slip zone in Figure 8. With the increase of the load *F*, both the shaft and the shaft sleeve will deform, resulting in the separation of the small gap between them, corresponding to the open area in Figure 8, and the normal and tangential contact stresses will decrease in the region.

**Figure 9:**
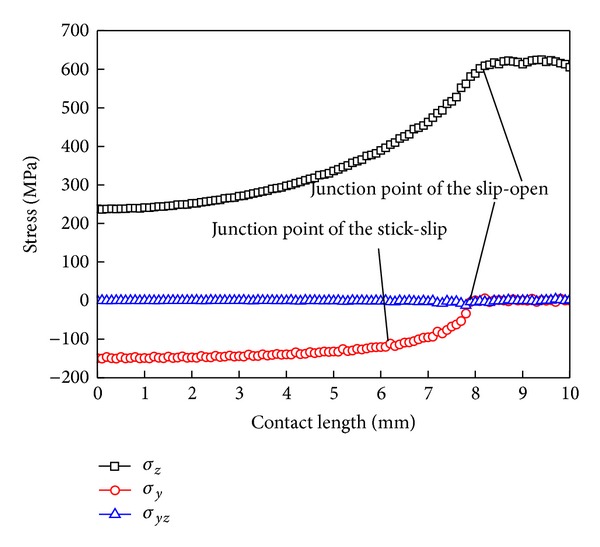
The stress distribution along the contact line.

By taking the line AB on the contact surface for analysis, Figure 9 shows the tensile stress *σ*_*z*_, compressive stress *σ*_*y*_, and shear stress *σ*_*yz*_. In the stick zone, the tensile stress is gradually increasing from left to right, the compressive stress is gradually reduced, and the shear stress is nearly 0. Mutations of the three stresses occur in the stick-slip at the junction point. In the slip zone, the changing rates of stresses are all increased. At the junction point of slip-open, the shear stress reaches its maximum. In the open area, the tensile stress reaches its maximum, but the compressive stress and shear stress are both 0.

## 6. Key Parameters' Effects on Fretting Wear

### 6.1. Bending Loads

**Figure 10:**
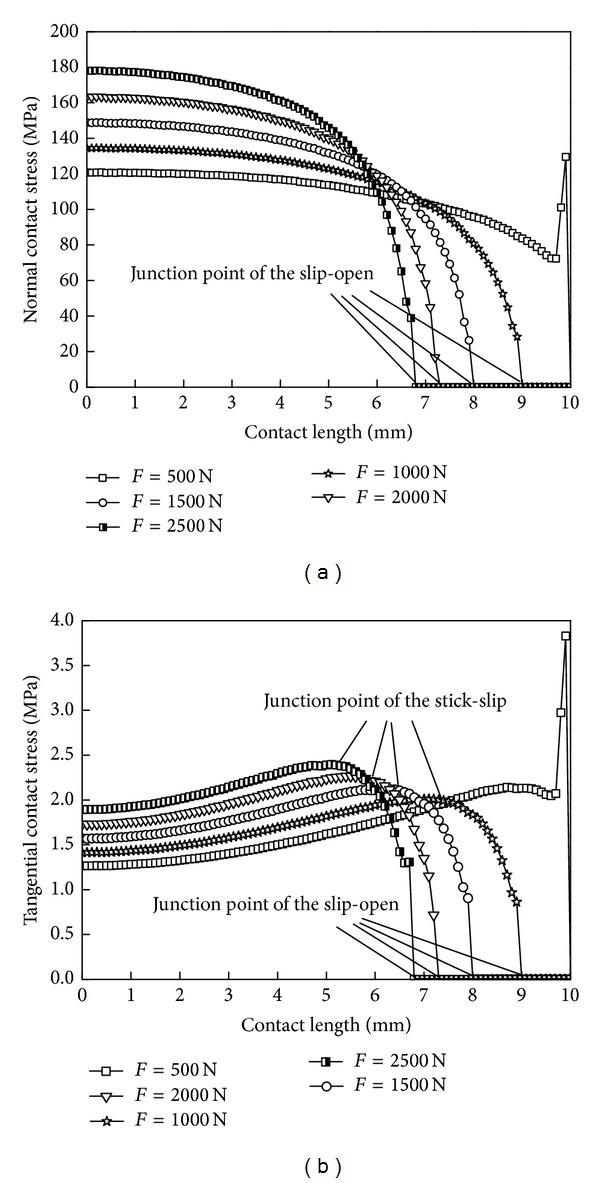
Contact stress distribution curves under different loads. (a) Normal contact stress and (b) tangential contact stress.

Figure 10 shows the contact stress along the contact line, when the maximum bending load is varied. When the load is small, the contact area is completely in the stick state, and the singularity of the contact stress exists in the end. When the load is gradually increasing, a relative sliding occurs in the contact zone, and slip area and open area will appear, while stick zone gets smaller. In the stick zone, the normal contact stress along the axial direction decreases gradually and reduces to 0 at the junction point of the slip-open. Tangential contact stress along the axial direction increases gradually in the stick zone and reaches its maximum at the junction point of the stick-slip. It begins to decrease in the slip zone; at last, it reduces to 0 in the open zone. As the load increases, the normal and tangential contact stress in the stick zone both increase.

Table 1 shows the sizes of the three zones under different loads. As the load increases, the correction factor *k* is gradually getting smaller, but it has nonlinear changes. Stick zone gradually reduces, and the open zone gradually expends, while the slip zone remains basically unchanged, which shows that, in rotating bending loads, the size of the slip zones remains unchanged but moves back and forth along the axial direction as the loads' values vary.

The alternating tensile stress of the contact face controls the early expansion of fretting fatigue cracks; Figure 10 shows that the tensile stress reaches its maximum at the junction point of slip-open, and the fatigue crack easily sprouts here. Alternating shear stress is easy to make the contact surface appear; the plastic deformation and plastic alternating accumulation of damage is the root cause of the formation of cracks [10]. Because the shear stress at the junction point of slide-open has a mutation and the tensile stress even gets near the maximum, cracks are very easy to initiate and exacerbate.

### 6.2. Magnitude of Interference

**Figure 11:**
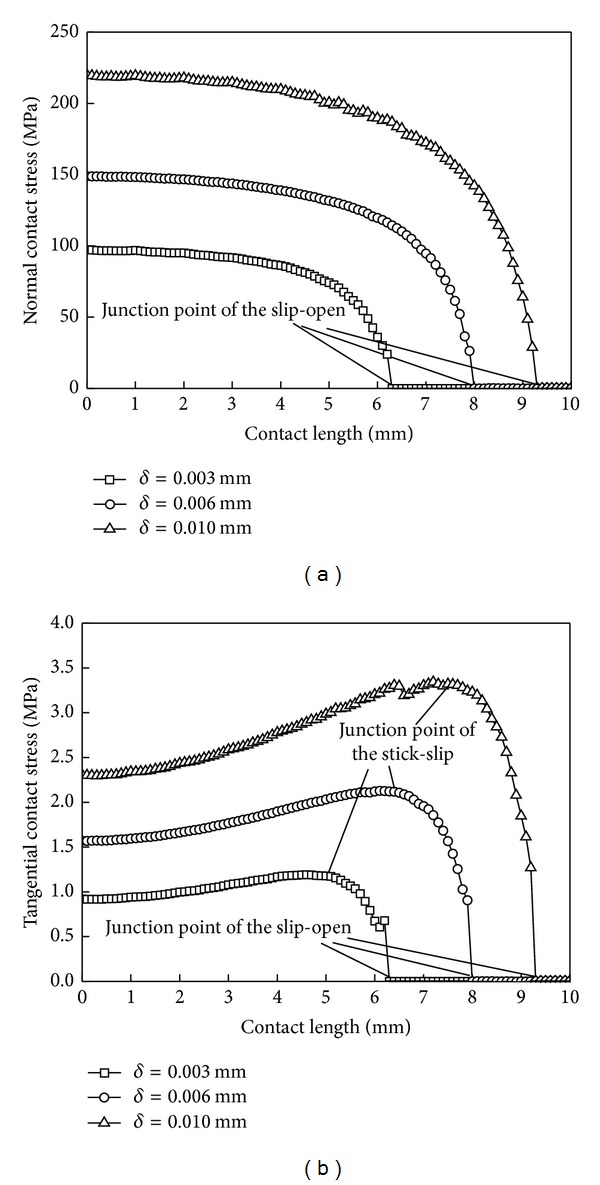
Contact stress distribution curves under different magnitudes of interference. (a) Normal contact stress and (b) tangential contact stress.

Figure 11 shows the contact stress distribution along the contact line with different interferences.  Table 2 shows the corresponding correction factors and contact zones' sizes with different interference. The greater the interference in the stick and slip zones is, the greater the tangential contact stress is. Although, according to the formula (6), in the linear elastic range, the contact stresses are proportional to the interferences, in the fretting contact, the contact stress and the interference are not a linear relationship. Only in the first half of the stick zone, the normal and tangential stresses are linearly proportional to the interferences.

Table 2 shows that the correction factor is also increased with the increase of the magnitude of interference. At the same time, the stick and slip zone gradually increase, while the open zone decreases gradually. The greater the interference value is, the smaller the wear zone becomes between the contact surfaces. The wear forms are mainly stick wear and fatigue wear. The tensile stress and shear stress will increase and lead to singular exacerbation and expansion.

### 6.3. Hollow Degree of Shaft

**Figure 12:**
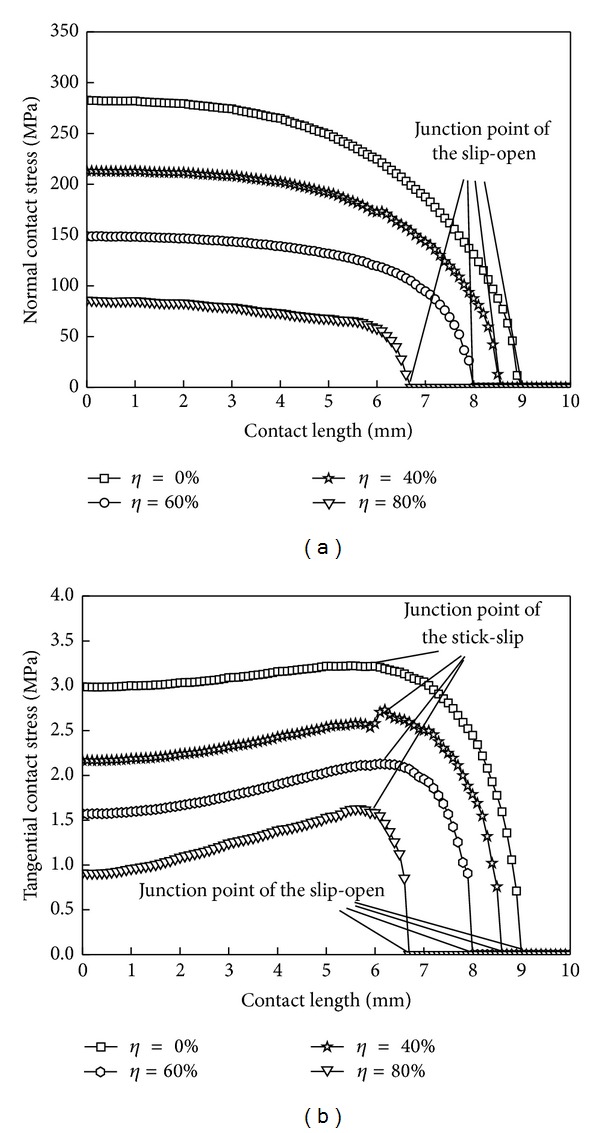
Contact stress distribution curves under different hollow degrees. (a) Normal contact stress and (b) tangential contact stress.

Figure 12 shows the normal and tangential contact stress distribution curves for different hollow degrees, and Table 3 shows the corresponding correction factor and the sizes of the three zones. The bigger the hollow degree is, the smaller the normal and tangential contact stresses are, but they have nonlinear changes. As the hollow degree increases, the correction factor also gets larger. The contact surface of the slip zone becomes smaller, and the open zone gets greater, but the stick zone has changed very little.

The above shows that the change of hollow degrees will influence the wear forms, but not the size of the wear region. And the increase of hollow degree would reduce the probability of occurrence of adhesive wear. The open area expansion will exacerbate the wear of the shaft sleeve end, and with the further increase of the amount of wear, the contact area of the shaft and shaft sleeve will reduce; the wear region will be extended to the stick zone at the same time.

### 6.4. Friction Coefficient

**Figure 13:**
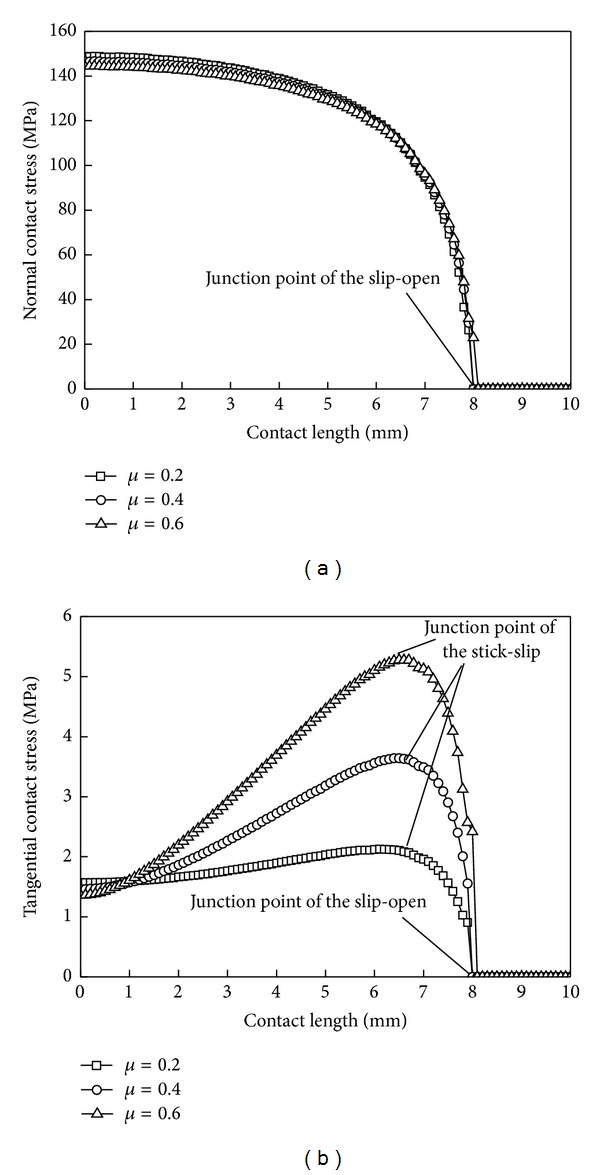
Contact stress distribution curves under different friction coefficients. (a) Normal contact stress and (b) tangential contact stress.

Figure 13 and Table 4 show the contact stress distribution and the contact zones' sizes under different friction coefficients. We know that friction coefficient has a small effect on the normal contact stress, but the tangential contact stress increases with the increase of friction coefficient. The position of junction point of the slip-open remains the same under different friction coefficients, which means friction coefficient does not affect the size of open zone. The junction point of the stick-slip moves outward, which means the stick zone increases gradually, and the slip zone decreases with the increase of friction coefficient. The above shows that the greater the friction coefficient is, the smaller the wear failure area becomes, and the crack area near the junction point of the stick-slip moves outwards gradually. With the increase of friction coefficient, the effects on correction coefficient decrease gradually.

### 6.5. Wear Area

**Figure 14:**
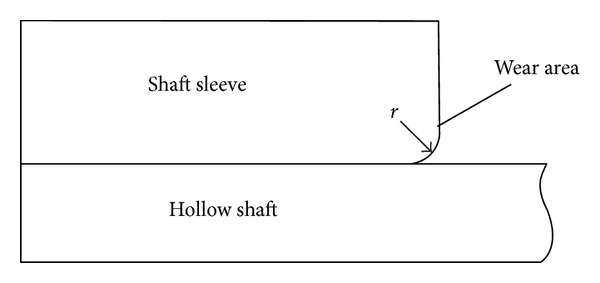
The wear schematic diagram of shaft sleeve.

In the end inner surface, shaft sleeve often appears to have serious wear due to larger alternating load. It was assumed that the collar end *r* varies to affect the wear to the microdynamic contact state and microfatigue crack initiation position in the paper.  Figure 14 shows the wear schematic diagram of shaft sleeve.

**Figure 15:**
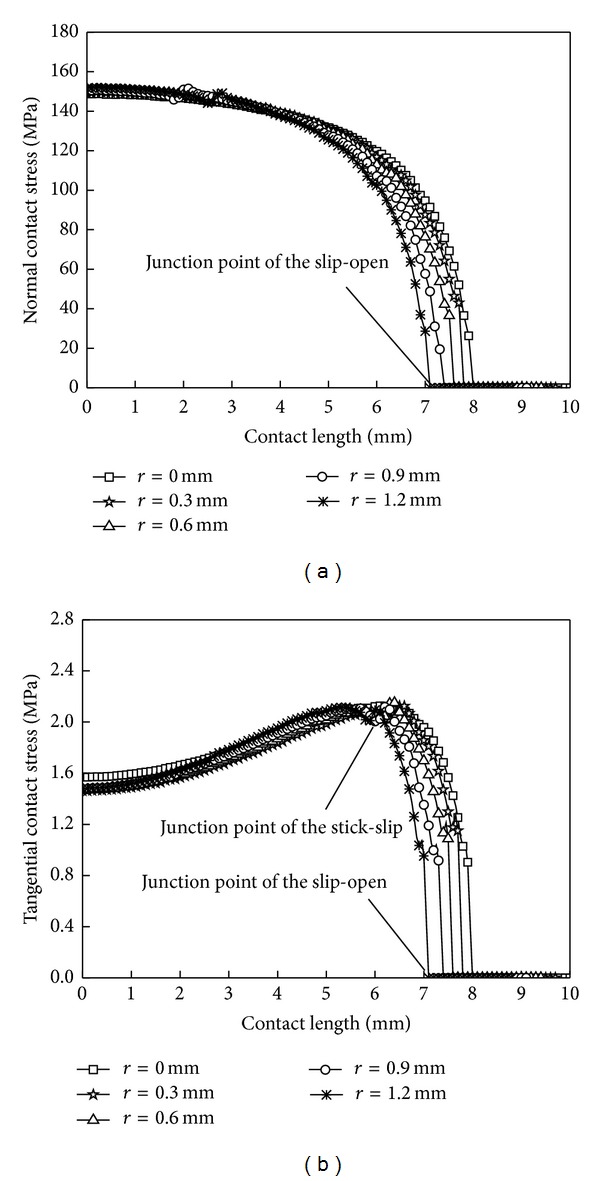
Contact stress distribution curves under different wear quantities. (a) Normal contact stress and (b) tangential contact stress.

When *r* is, respectively, 0 mm, 0.3 mm, 0.6 mm, 0.9 mm, and 1.2 mm, the contact stress distribution and contact zones' sizes are shown in Figure 15 and Table 5. In the stick zone, the contact stress on the line has a small change. In the slip zone, normal and tangential contact stresses reduce with the increase of the wear. The influence rule of wear quantity on the correction factor is not obvious. With the increase of wear quantity, the stick zone decreases, slip zone first increases and then decreases, open zone increases gradually, and they approximate to linear change, which illustrates that easy initiation microcrack area moves to contact center from both ends with the increase of working time in the fretting condition.

## 7. Conclusions

1. There is a stress concentration phenomenon in the inner surface collar end of shaft sleeve after interference assembly. The contact stress in the contact surface appeared “edge effect.” The greater the interference, the greater the normal contact stress. The greater the hollow degree of the shaft, the smaller the contact stress, but the “edge effect” is more obvious.
2. Correction formula can be used to determine the contact state and is suitable for fretting analysis of interference hollow shaft under the rotating bending load. The correction factor *k* is related to the interferences, hollow degrees, friction coefficients, and loads.
3. The stick zone, slip zone, and open zone obviously exist on the interference contact surface.
4. As the load increases, the stick zone gradually reduces; the open area is gradually expending, while the slip zone remains basically unchanged. The greater the magnitude of interference is, the greater the stick and slip zone are, and the smaller the open zone is. The bigger the hollow degree, the smaller the slip zone, and the greater the open zone, but stick zone has little change.

## Figures and Tables

**Table 1:** Sizes of three zones under different loads.

Load *F*/N	Correctness factor *k*	Stick zone *λ*_1_/mm	Slip zone *λ*_2_/mm	Open zone *λ*_3_/mm
1000	0.098	7.1	1.8	1.1
1500	0.090	6.1	1.8	2.1
2000	0.085	5.4	1.8	2.8
2500	0.083	5.1	1.6	3.3

**Table 2:** Sizes of three zones under different interferences.

Interference *δ*/mm	Correction factor *k*	Stick zone *λ*_1_/mm	Slip zone *λ*_2_/mm	Open zone *λ*_3_/mm
0.003	0.074	4.6	1.6	3.8
0.006	0.090	6.1	1.8	2.1
0.010	0.099	7.2	2.0	0.8

**Table 3:** Sizes of three zones under different hollow degrees.

Hollow degree *η*	Correction factor *k*	Stick zone *λ*_1_/mm	Slip zone *λ*_2_/mm	Open zone *λ*_3_/mm
0%	0.068	5.5	3.4	1.1
40%	0.080	6.2	2.3	1.5
60%	0.090	6.1	1.8	2.1
80%	0.128	5.7	0.9	3.4

**Table 4:** Sizes of three zones under different friction coefficients.

Friction coefficient *μ*	Correction factor *k*	Stick zone *λ*_1_/mm	Slip zone *λ*_2_/mm	Open zone *λ*_3_/mm
0.2	0.090	6.1	1.8	2.1
0.4	0.083	6.5	1.4	2.1
0.6	0.083	6.7	1.3	2.0

**Table 5:** Sizes of three zones under different wear quantities.

Wear quantity *r*/mm	Correction factor *k*	Stick zone *λ*_1_/mm	Slip zone *λ*_2_/mm	Open zone *λ*_3_/mm
0	0.090	6.1	1.8	2.1
0.3	0.085	5.7	2.0	2.3
0.6	0.087	5.6	1.9	2.5
0.9	0.090	5.6	1.7	2.7
1.2	0.089	5.3	1.7	3.0
